# Reduced overtriage is associated with increased patient flow at pediatric emergency department

**DOI:** 10.1186/s13049-026-01573-w

**Published:** 2026-01-31

**Authors:** Hannah Sjöstedt, Samah Habbouche, Lina Holmqvist, Jimmy Celind

**Affiliations:** 1 Gothenburg Emergency Medicine REsearch Group (GEMREG), The Sahlgrenska University Hospital, Gothenburg, Sweden; 2 Department of Pediatrics, Institute of Clinical Sciences, The Sahlgrenska Academy, University of Gothenburg, Gothenburg, Gothenburg, Sweden; 3 Department of Internal Medicine and Clinical Nutrition, Institute of Medicine, The Sahlgrenska Academy, Gothenburg University, Gothenburg, Sweden; 4 Center for Bone and Arthritis Research, Institute of Medicine, The Sahlgrenska Academy, University of Gothenburg, Gothenburg, Sweden

**Keywords:** Pediatric emergency medicine, Emergency medicine, Triage, Crowding

## Abstract

**Background:**

Crowding is the major threat to patient safety at pediatric emergency departments (PED) and a slow patient flow is its main contributor. Overtriage may impair patient flow, requiring triage systems to balance undertriage-overtriage risks. To reduce overtriage, RETTS-p was replaced by WEST-P at Gothenburg’s PED in 2021. No prior studies compare pediatric triage systems’ impact on patient flow. Our objective was to compare patient flow from different triage systems with different levels of overtriage.

**Methods:**

This retrospective, observational study was performed at Queen Silvia PED in Gothenburg, Sweden. Triage urgency and patient flow metrics were collected in March 2018, 2019 (RETTS-p), 2022 and 2023 (post-WEST-P implementation). All triaged patients assessed by a physician were included. Patient inflow and the number of health care personnel were similar across the four months.

**Results:**

The study population (*N* = 8,125) included the RETTS-p group (*N* = 4,129) and the WEST-P group (*N* = 3,996). 21% of patients in WEST-P and 30% in RETTS-p were high urgency (red or orange). The median time to physician was 4 min shorter for red urgency patients, and 18 min shorter for orange urgency patients (both *p* < 0.001) in WEST-P compared to RETTS-p, from 5 to 1 and 30 to 12 min respectively. The median length of stay for all patients was 23 min shorter with WEST-P compared to RETTS-p, 166 and 189 min respectively (*p* < 0.001).

**Conclusion:**

Less overtriage can shorten time to physician for high urgency patients and reduce the length of stay for patients of all priorities at a PED.

**Supplementary Information:**

The online version contains supplementary material available at 10.1186/s13049-026-01573-w.

## Background

Emergency department (ED) crowding occurs when demand exceeds available resources and represents a major threat to patient safety worldwide [1, 2]. In pediatric emergency departments (PEDs), prolonged length of stay (LOS) is a key contributor to crowding, and the triage system in use is believed to play a significant role in determining patient flow [3]. Triage systems prioritize patients based on the urgency of their medical condition, thereby allocating resources to those in most urgent need of treatment [4]. Undertriage, assigning a lower priority than medically warranted, poses a direct risk to patient safety [5]. Overtriage, in contrast, occurs when patients are given a higher priority than required. While overtriage does not endanger the individual patient, it consumes valuable ED resources, potentially resulting in delayed care for truly urgent patients and increased interruptions in clinical workflows. Prior studies have shown that frequent interruptions impair physicians’ cognitive performance and raise the risk of medical errors [6, 7]. Overtriage is therefore likely to contribute to increased LOS in PEDs. Structured pediatric triage has been associated with improved patient flow [8]. Consequently, triage system design may have a substantial impact on patient flow, ED crowding, and associated risks. However, to our knowledge, no studies have compared patient flow between different triage systems.

Rapid Emergency Triage and Treatment Systems – pediatrics (RETTS-p) is the most widely used pediatric triage system in Scandinavia [9]. It is a five-level triage system, its red and orange triage categories denote high urgency, yellow medium, and green and blue low urgency. RETTS-p is characterized by a high degree of overtriage and therefore limited precision [10]. To improve precision, the PED at Queen Silvia Children’s hospital in Gothenburg, Sweden replaced RETTS-p with the locally developed West Coast System for Triage—Pediatric (WEST-P) in 2021. WEST-P, a four-level triage system, designates red and orange as high urgency, similar to RETTS-p. Compared to RETTS-p, previous studies show that WEST-P reduces the proportion of patients assigned high urgency and overtriage while maintaining undertriage at a low and safe level [11, 12].

We hypothesize that the higher precision of WEST-P, with its lower rate of overtriage, results in shorter time to physician for high urgency patients compared to RETTS-p. Furthermore, by reducing unnecessary interruptions for clinicians, WEST-P may facilitate more efficient workflows, ultimately shortening LOS for all patients.

### Aim

The aim of this study was to investigate whether a more precise triage system, characterized by lower overtriage, improves time to physician for high urgency patients and reduces LOS for all patients at a PED.

## Method

### Study design and setting

This retrospective observational study was conducted at the PED of Queen Silvia Children’s Hospital, within the Sahlgrenska University Hospital system in Gothenburg, Sweden. The study is part of the research initiative “State of the Pediatric Emergency Department” (SPEED). The PED is the largest in the Nordic region, receiving approximately 60,000 annual visits from patients aged 0–15 years. It is the sole PED serving the greater Gothenburg area and acts as the region’s level 1 trauma center. All pediatric emergency cases are directed to this department, regardless of etiology. The PED has a fast-track to a lower level of care for patients with pre-defined non-emergent conditions such as mild obstructive episode or suspected non-displaced fracture. Human Ethics and Consent to participate declaration: The study was approved by the Swedish Ethical Review Authority (application number 2025-00551-02), who waived the requirement for informed consent, in accordance with the Swedish Ethical Law (2003:460).

### Participants

The study included four months of data from March 2018 and 2019 (pre-pandemic, using RETTS-p), and March 2022 and 2023 (post-pandemic, using WEST-P). These periods were selected to minimize confounding from the COVID-19 pandemic and to represent the high-inflow infectious season. The COVID-19 pandemic was excluded as the inflow of patients was halved, and infectious related visits were nearly absent at the PED during this period. All PED patients during the study months were eligible for inclusion if they 1) underwent full triage and 2) were assessed by a physician. Patients were excluded if they lacked a triage category or assessment by physician in the patient administrative system, indicating that they were either not triaged and/or not assessed by a physician. Patients directed to the fast-track were excluded, as they typically do not receive full triage or physician assessment. The study was approved by the Swedish Ethical Review Authority, who waived the requirement for informed consent.

### Data

Data were extracted from the administrative systems Cognos (IBM Corp., Armonk, NY, USA) and Elvis (Swissmedic, Bern, Switzerland). Variables included triage urgency (color), age, sex, reason for visit, and four time points: 1) arrival at PED, 2) triage completion, 3) initial physician assessment, and 4) discharge from the PED.

### Measurements

Data from March 2018 and 2019 were pooled as the RETTS-p group, and data from March 2022 and 2023 as the WEST-P group. Age and sex distribution were assessed across groups. Administrative categories for reason for visit changed during the COVID-19 period, limiting comparability (Supplementary Table 1).

### Primary outcomes

Two patient flow metrics were compared between the RETTS-p and WEST-P groups. For high urgency patients (red or orange triage), the primary outcome was time to physician, measured from triage completion to initial physician assessment. For all patients, LOS was defined as the time from PED arrival to discharge.

### Analysis

Categorical variables are presented as frequencies, and continuous non-normally distributed variables as medians with interquartile ranges (IQRs). Group differences for categorical variables were tested using chi-square tests; for continuous variables, Mann–Whitney U tests were used. Linear and quantile (median) regression were used to analyze the association between daily numbers of high-priority patients and LOS. A *p*-value < 0.05 was considered statistically significant. Analyses were performed using SPSS version 29 (IBM Corp., Armonk, NY, USA).

### Sensitivity analyses

To examine whether differences in patient flow persisted during low-inflow periods, a sensitivity analysis analogous to the analyses of March was conducted using data from September 2018, 2019, 2022, and 2023.

To assess the impact of age distribution on LOS, each March dataset was analyzed by age group (< 1 year vs. ≥ 1 year). If age-specific LOS was similar within a year but LOS differed across years, this would suggest that age distribution alone did not explain LOS differences.

Additionally, LOS was analyzed independently by month, and linear regression was performed to assess the association between the daily number of high urgency patients and median LOS. For the month-stratified sensitivity analysis, linear regression was performed, and residual-versus-fitted and Q–Q plots were inspected, showing no major deviations. Because LOS is inherently skewed, we additionally conducted quantile (median) regression as a robustness analysis, using quantile regression.

#### Language editing

The final language editing of this manuscript was supported by an AI-based language model (ChatGPT, OpenAI) to improve clarity, grammar, and scientific phrasing. All content was reviewed and approved by the authors.

## Results

### Characteristics of the study subjects

**Fig. 1:**
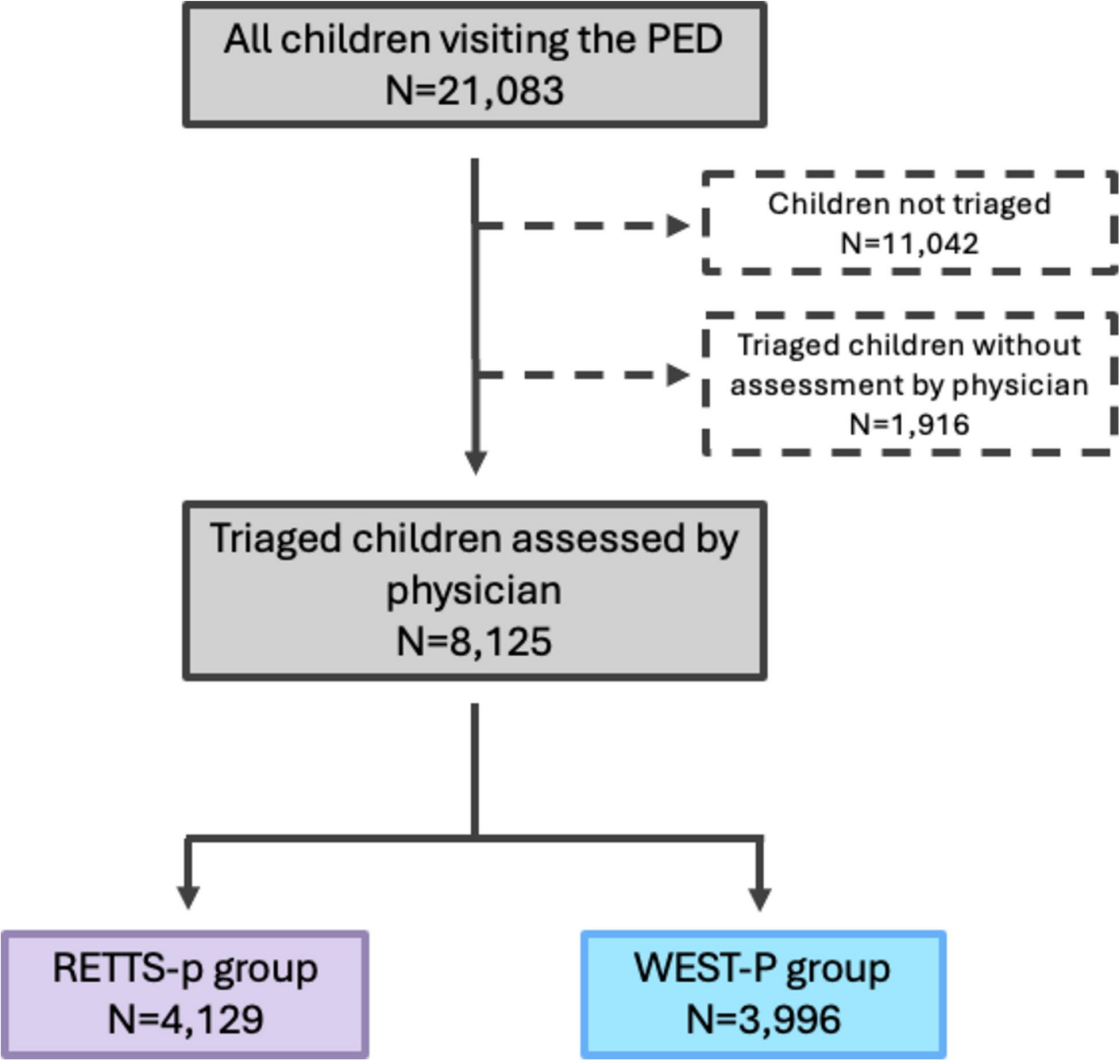
Inclusion of participants. Flow chart depicting the inclusion of participants to the study population, and the grouping into Rapid Emergency Triage and Treatment – pediatrics (RETTS-p) and West Coast System for Triage – Pediatrics (WEST-P) groups

Patient inflow and staffing levels were consistent across all study months. Of 21,083 total PED visits, 8,125 patients met inclusion criteria (Fig. 1): 4,129 in the RETTS-p group and 3,996 in the WEST-P group (Table 1).

**Table 1:** Differences in sex and age

	**2018**	**2019**	**2022**	**2023**	**RETTS-p**	**WEST-P**
*Participants*	2078	2051	1946	2050	4129	3996
*Females (%)*	929 (44.7)	899 (43.8)	874 (44.9)	957 (46.7)	1,828 (44.3)	1,831 (45.8)
*Median age, years (IQR)*	3.5 (0.8–9.9)	3.6 (0.8–9.8)	4.6 (1.3–11.0)	5.3 (1.6–10.9)	3.5 (0.8–9.8)	5.0*** (1.5–11.0)
*Patients* < *1 year (%)*	567 (27.3)	550 (26.8)	419 (21.5)	370 (18.0)	1,117 (27.0)	789*** (19.7)

The differences in sex and age between the months of March of the different years, and groups

***p < 0.001 compared to RETTS-p

**Fig. 2:**
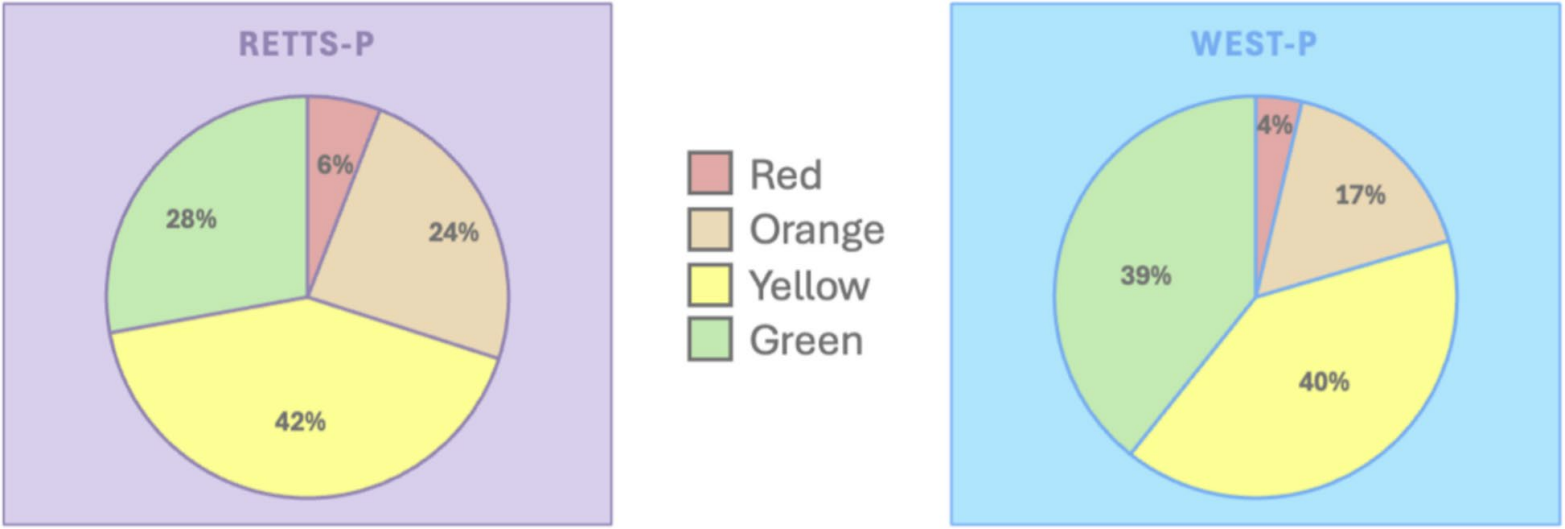
Distribution of triage urgency**.** Showing the distribution of triage urgency in the RETTS-p and WEST-P groups. All differences between the two groups are statistically significant. *P* < 0.001 between the red, orange and green urgencies separately, and *p* < 0.05 between the yellow urgencies

The median age was 3.5 years in the RETTS-p group and 5.0 years in the WEST-P group, primarily due to a higher proportion of infants (< 1 year) in the RETTS-p group. The proportion of high urgency triaged patients (red and orange) was significantly lower in the WEST-P group than in the RETTS-p group (21% vs. 30%, *p* < 0.001; Fig. 2).

Within the RETTS-p group, 2019 had a lower proportion of high urgency patients than 2018: 106 (5%) red patients and 471 (23%) orange patients compared to 137 (7%) and 523 (25%) respectively, *p* < 0.05.

### Main results

**Fig. 3:**
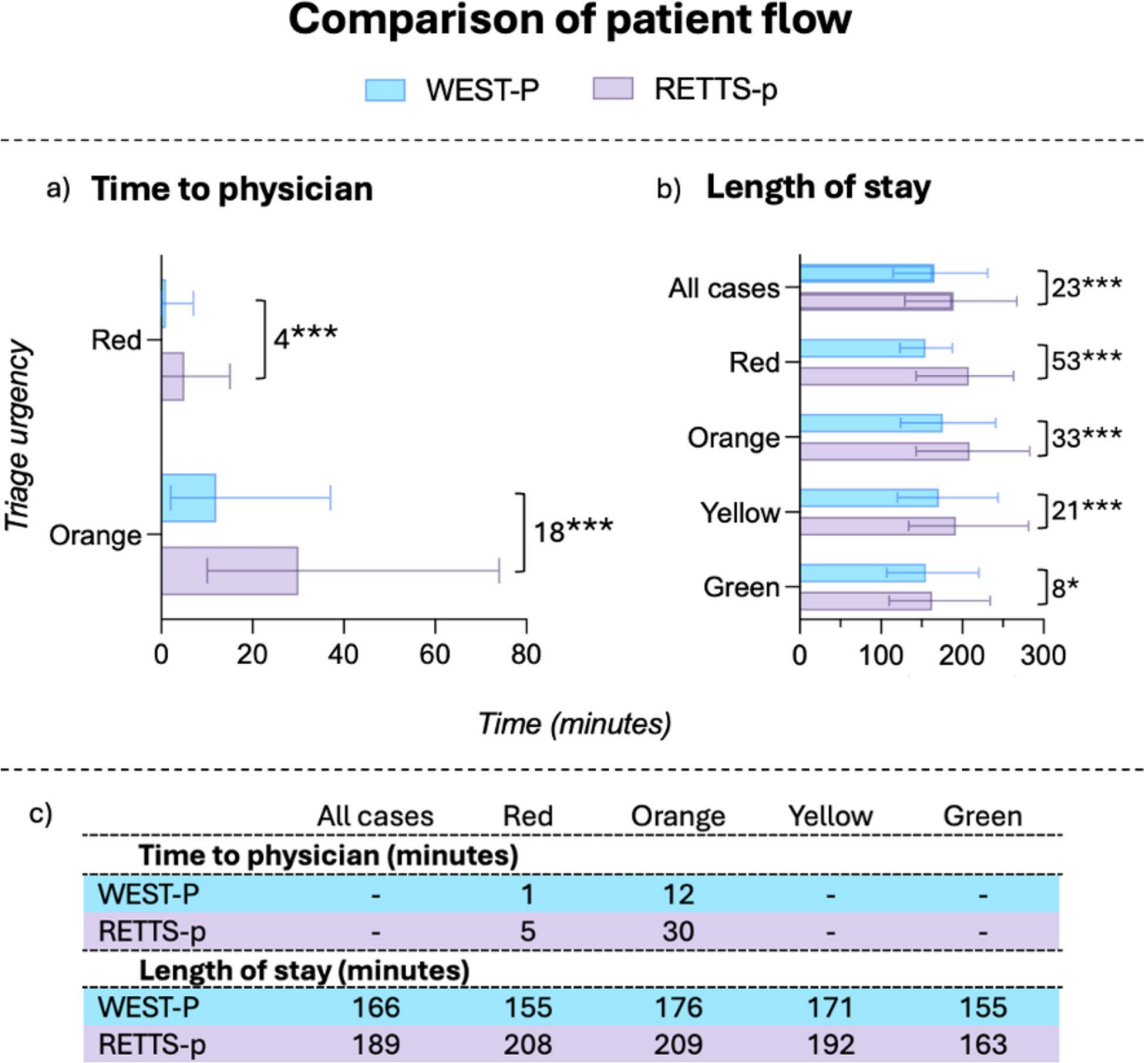
Comparison of patient flow. Showing differences in a) time to physician; and b) length of stay depending on triage urgency. Bars represent median, and whiskers represent interquartile range. Also showing c) median values of the patient flows within each group. Differences between WEST-P and RETTS-p shown in minutes, *p < 0.05, ***p < 0.001

Among high urgency patients, the WEST-P group had significantly shorter time to physician: a 4 min reduction for red urgency (*p* < 0.001) and 18 min for orange (*p* < 0.001) (Fig. 3). Overall, median LOS was 23 min shorter in the WEST-P group (*p* < 0.001), with the most prominent reduction, 53 min, for red urgency patients (Fig. 3).

### Sensitivity analyses

The September analysis during low inflow confirmed the main findings: median time to physician was 5 min shorter for red and 10 min for orange urgency patients with WEST-P (*p* < 0.001). Median LOS was 6 min shorter overall and 44 min shorter for red urgency patients (*p* < 0.001).

In the age-based analysis, LOS did not differ significantly by age (< 1 vs. ≥ 1 year) within 2019 and 2022 individually. However, LOS in 2022 was 13 min shorter than in 2019 (*p* < 0.001), suggesting age distribution did not solely explain group differences.

Linear regression on RETTS-p data from 2018 and 2019 showed that each additional high urgency patient was associated with a 2.7-min increase in daily median LOS (*p* < 0.001). Across the full dataset, the corresponding increase was 2.8 min (*p* < 0.001). In the sensitivity analysis using quantile (median) regression, the association remained significant: each additional high-urgency patient was linked to a 2.3-min increase in median LOS (95% CI 1.8–2.8), consistent with the primary linear regression and supporting the robustness of the results.

## Discussion

In this retrospective observational study of over 8,000 PED visits at Queen Silvia Children’s Hospital, the patient flow from two different triage system was evaluated. The implementation of WEST-P was associated with significantly shorter time to physician for high urgency patients and shorter LOS for all patients, compared to RETTS-p. Improvements were most notable during high-inflow months but remained statistically significant during periods of lower patient volumes.

Although no prior studies have compared triage systems directly in terms of patient flow, the effects of structured triage have been documented. In China, structured triage implementation reduced overall wait times by 4.3 min [8]. Other studies have reported broader impacts: in Guatemala, implementation of structured triage reduced hospital and ICU admissions [13], and in Kenya, child mortality declined following hospital reforms including triage implementation [14]. However, the multifactorial nature of those interventions limits causal effect, and the low pediatric mortality rate in Sweden renders such outcomes impractical to assess [15]. Importantly, structured triage was already in place at our study site prior to WEST-P.

Kula et al. emphasized the role of triage precision in optimizing workflow and mitigating crowding in PEDs [3]. Efficient triage not only identifies patients in urgent need of care [5] but also reduces system strain, benefiting even those with less severe conditions. Our findings support this: during high-inflow seasons, WEST-P significantly reduced LOS across all triage urgencies, suggesting a system-wide benefit.

Although the observational nature of this study cannot determine causal mechanisms, our hypothesis, that reduced overtriage leads to fewer workflow interruptions and improved efficiency, is consistent with previous findings linking interruptions to impaired ED performance [6]. Notably, our regression analyses showed that an increased number of high urgency patients prolonged LOS, regardless of the triage system used.

Limitations include the use of different study populations and time periods for RETTS-p and WEST-P, separated by three years and a pandemic. While no major structural changes were made to the PED aside from the triage system change, unmeasured confounders such as staff turnover or heightened focus on efficiency may have influenced results. Although results were robust during periods of both high and low inflow, they may not be generalizable across all seasons. Moreover, time stamps were manually recorded, introducing potential measurement error; however, such error is likely non-differential. While age distribution differed between groups, our sensitivity analyses suggest this did not drive LOS differences.

Strengths of the study include its large, real-world cohort and the use of population-based, operational data. To our knowledge, this is the first study demonstrating that triage system precision significantly affects both time to physician and LOS for PED patients.

In summary, the previously established more precise WEST-P triage system was associated with reduced time to physician for high urgency patients and significantly shorter LOS for all patients at a high-volume pediatric emergency department. These improvements may mitigate crowding and enhance patient safety. Further research is warranted to confirm these findings and expose the underlying mechanisms.

## Supplementary Information

Supplementary Material 1.

## Data Availability

No datasets were generated or analysed during the current study.

## Abbreviations

PED: Pediatric emergency department
RETTS-p: Rapid Emergency Triage and Treatment Systems – Pediatrics
WEST-P: West Coast System for Triage - Pediatric
ED: Emergency department
LOS: Length of stay
IQR: Interquartile range

## References

[1] Javidan AP, Hansen K, Higginson I, Jones P, Lang E, Ifem Task Force on Emergency Department Crowding AB. The International Federation for Emergency Medicine report on emergency department crowding and access block: a brief summary. Can J Emerg Med. 2021;23(1):26–8. 10.1007/s43678-020-00065-9. 10.1007/s43678-020-00065-9 PMC7807403 33683618

[2] Devia Jaramillo G, EsmeralZuluaga N, Velandia Avellaneda VA. Effective strategies for reducing patient length of stay in the emergency department: a systematic review and meta-analysis. BMC Emerg Med. 2025;25(1):25. 10.1186/s12873-024-01163-y. 39979831 10.1186/s12873-024-01163-y PMC11841229

[3] Kula R, Popela S, Klucka J, Charwatova D, Djakow J, Stourac P. Modern Paediatric Emergency Department: Potential Improvements in Light of New Evidence. Children (Basel). 2023;10(4). 10.3390/children10040741. 10.3390/children10040741 PMC10137157 37189990

[4] Kuriyama A, Urushidani S, Nakayama T. Five-level emergency triage systems: variation in assessment of validity. Emerg Med J. 2017;34(11):703–10. 10.1136/emermed-2016-206295. 28751363 10.1136/emermed-2016-206295

[5] Berkowitz D, Cohen JS, McCollum N, Rojas CR, Chamberlain JM. Delays in treatment and disposition attributable to undertriage of pediatric emergency medicine patients. Am J Emerg Med. 2023;74:130–4. 10.1016/j.ajem.2023.09.054. 37826993 10.1016/j.ajem.2023.09.054

[6] Weigl M, Catchpole K, Wehler M, Schneider A. Workflow disruptions and provider situation awareness in acute care: an observational study with emergency department physicians and nurses. Appl Ergon. 2020;88:103155. 10.1016/j.apergo.2020.103155. 32678775 10.1016/j.apergo.2020.103155

[7] Westbrook JI, Raban MZ, Walter SR, Douglas H. Task errors by emergency physicians are associated with interruptions, multitasking, fatigue and working memory capacity: a prospective, direct observation study. BMJ Qual Saf. 2018;27(8):655–63. 10.1136/bmjqs-2017-007333. 29317463 10.1136/bmjqs-2017-007333 PMC6204927

[8] Lin GX, Yang YL, Kudirka D, et al. Implementation of a pediatric emergency triage system in Xiamen, China. Chin Med J (Engl). 2016;129(20):2416–21. 10.4103/0366-6999.191755. 27748332 10.4103/0366-6999.191755 PMC5072252

[9] AB P. RETTS. Predicare AB. 20230508, 2023. https://predicare.com/sv/om-retts/. Accessed 20230508, 2023.

[10] Magnusson C, Herlitz J, Karlsson T, Jimenez-Herrera M, Axelsson C. The performance of the EMS triage (RETTS-p) and the agreement between the field assessment and final hospital diagnosis: a prospective observational study among children < 16 years. BMC Pediatr. 2019;19(1):500. 10.1186/s12887-019-1857-0. 31842832 10.1186/s12887-019-1857-0 PMC6912993

[11] Sjostedt H, Kindblom JM, Celind J. A low proportion of undertriage validates the new West coast system for triage-Paediatric. Acta Paediatr. 2024. 10.1111/apa.17107. 10.1111/apa.17107 38235600

[12] Sjostedt H, Leo Lemarquis A, Klasson M, Holmqvist L. [Fewer overtriaged children with PEPP compared to RETTS-p at a Swedish pediatric emergency department]. *Lakartidningen*. 2022;119:21237. Svenskanpassad triage gav farre overprioriteringar pa barnakut. https://www.ncbi.nlm.nih.gov/pubmed/35875906. 35875906

[13] Crouse HL, Torres F, Vaides H, et al. Impact of an emergency triage assessment and treatment (ETAT)-based triage process in the paediatric emergency department of a Guatemalan public hospital. Paediatr Int Child Health. 2016;36(3):219–24. 10.1179/2046905515Y.0000000026. 25940386 10.1179/2046905515Y.0000000026

[14] Ayieko P, Ntoburi S, Wagai J, et al. A multifaceted intervention to implement guidelines and improve admission paediatric care in Kenyan district hospitals: a cluster randomised trial. PLoS Med. 2011;8(4):e1001018. 10.1371/journal.pmed.1001018. 21483712 10.1371/journal.pmed.1001018 PMC3071366

[15] Unicef country profiles: Sweden. Unicef. 250407, 2025. https://data.unicef.org/country/swe/. Accessed 250704, 2025.

