# Supplementary material for: Reduced overtriage is associated with increased patient flow at pediatric emergency department
Source: Scand J Trauma Resusc Emerg Med. 2026 Jan 31;34:47. doi: 10.1186/s13049-026-01573-w (PMC12930879; doi:10.1186/s13049-026-01573-w)
Supplement: Supplementary file 1 — Supplementary Material 1. [file 13049_2026_1573_MOESM1_ESM.pdf]

Reason for PED visit	2018	2019	2022	2023	All years
Abdominal pain	174	194	160	187	715
Abscess	6	13			19
Allergy	19	26	23	19	87
Amputation	1				1
Anal		1			1
Anemia	2	2			4
Anorectal		1			1
Apnoea	2	2			4
Appendicitis		1			1
Arrythmia		1			1
Ashtma	1	4			5
Back pain	4	7		1	12
Bite	1	1	1	1	4
Burn	21	8	20	9	58
Chest pain	5	9	11	16	41
Common cold	38	63			101
Commotio	15	7			22
Constipation	4	7			11
Convulsions	61	36	46	58	201
Cough	24	33			57
Croup	13	9			22
Crush injury to finger/toe	12	10	17	23	62
Crying baby	11	15	18	7	51
Diabets	7	7	13	14	41
Diarrhoea	5	3	12	7	27
Dyspnoea	353	312	52	118	835
Ear infection		1			1
Ear issue	6	12	1	10	29
Electric accident	1				1
Exanthema	8	21	11	19	59
Eye problem	10		12	9	31
Febrile convulsion	7	6			13
Feet issue	1				1
Fever	236	203		109	548
Foreign body	16	18	21	18	73
Gastroenteritis	11	13			24
Genital issue	1	4	10	11	26
Headache	11	13	14	10	48
Heat disease	3	2	2	6	13
Hematemesis	2		3	4	9
Hernia	5	2	6	1	14
Hip issue	1				1
Hip pain	3	10			13
Infection	80	55			135
Intoxication	6	13	26	15	60
Issue with feeding tube	1	1			2
Itchiness	1				1
Knee	28	56	88	65	237
Limb injury	311	298	285	269	1163
Limping	7	14	21	32	74
Local infection	13	9	19	31	72
Luxation		1			1
Melena	7	4	9	8	28
Mental illness	1		6	13	20
Nail issue		3			3

Nausea	54	43	124	60	281
Neck problem	10	7	5	5	27
Neurological issue	11	7	9	11	38
Observation	1				1
Other	5	5			10
Pain	6	9	9	25	49
Penis issue	14	10			24
Physical abuse	6	13	7	11	37
Pneumonia	3	15			18
Poor weight gain		7	10	2	19
Post operative complication	3	2	9	8	22
Problem with cast	1	1	4	12	18
Return visit	32	39			71
Scrotal pain	35	29	35	42	141
Shunt issue	2	4			6
Skin issue	6	2			8
Skull condition	22	19			41
Sore throat	4	6			10
Spots on skin	3				3
Stomach issue	9	9			18
Symptoms related to covid-19 pandemic			380	310	690
Trauma	4	3			7
Trauma level 1			1	1	2
Trauma level 2			8	3	11
Trauma level 3			17	7	24
Trauma on the street	13	9	13	11	46
Trauma to nose	5		1		6
Trauma to skull	29	36	66	86	217
Trauma to thorax/abdomen	2	4	1		7
Traumatic fall	26	36	69	58	189
Triage			1		1
Umbilical hernia	1				1
Unspecified	145	127	206	229	707
Upper respiratory tract infection	1	1		2	4
Urinary problem	16	25	24	34	99
Urinary tract infection	2	1			3
Wound	31	24	40	43	138
Wound dressing	1				1
Wound problem	19	27			46
Sum of all reasons	**2078**	**2051**	**1946**	**2050**	**8125**
